# Genome-wide association analysis identifies SNP loci for multi-stage yield-related traits in wheat

**DOI:** 10.3389/fpls.2026.1807558

**Published:** 2026-06-03

**Authors:** Mengyao Li, Ran He, Qiangqiang Chai, Xue Wang, Yijia Fu, Ruimei Liu, Yan Zhao

**Affiliations:** State Key Laboratory of Wheat Improvement, College of Agriculture, Shandong Agricultural University, Tai’ an, Shandong, China

**Keywords:** agronomic traits, genome-wide association, SNP, wheat, yield

## Abstract

**Introduction:**

Wheat is a major global food crop, and improving its yield is essential for food security. Identifying genetic loci and candidate genes associated with yield-related traits is crucial for molecular breeding.

**Methods:**

A panel of 768 common wheat varieties from domestic and international sources was evaluated over two consecutive years (2024 and 2025) in Tai’an. Three growth-stage traits, eight agronomic traits, and three seedling-stage traits, including root length, seedling weight, and seedling height, were measured. Genotyping was performed using a 55K SNP array, and a genome-wide association study was conducted using a linear mixed model.

**Results:**

Population structure and kinship analyses classified the varieties into primitive landraces and modern cultivars. A total of 49,768 high-quality SNPs were identified, covering 82.73% of the genome with an average marker density of 0.29 Mb. Among them, 17,293, 18,313, and 14,162 SNPs were located on the A, B, and D subgenomes, respectively. Association analysis revealed 544 SNP loci significantly associated with yield-related traits across 21 chromosomes, of which 49 were consistently detected in both years. Additionally, 60 loci were associated with two or more traits, indicating potential pleiotropic effects, and were distributed on 16 chromosomes (excluding 3D, 4B, 4D, 7A, and 7B).

**Discussion:**

This study identifies SNP markers associated with key traits across growth stages in common wheat and provides a basis for the discovery of candidate genes and favorable alleles. These findings may facilitate the utilization of elite genetic resources for yield improvement in wheat breeding.

## 1 Introduction

By the mid-21st century, the global population is projected to reach 9 billion, accompanied by rising demand for healthy and nutritious food, which will intensify pressure on agricultural production worldwide (Long et al., 2015; Massawe et al., 2016; Ammar et al., 2023). Wheat provides nearly one-fifth of global calorie and protein intake and therefore plays a central role in food security (Zheng et al., 2025). Projections indicate that by 2050, assuming no expansion of arable land, wheat yields must increase annually by 2.0% to meet basic food requirements. However, current global agricultural productivity growth remains below 1.7%, creating a substantial challenge to future food security (Brisson et al., 2010; Iizumi et al., 2021; Zhang et al., 2026a; De Paola et al., 2025).

Most wheat yield-related traits are quantitative in nature, controlled by multiple genes and governed by complex regulatory networks. High−efficiency genome-wide association study (GWAS) enables systematic identification of functional genes underlying key agronomic and quality traits, thereby providing a theoretical basis for breeding high-yielding, high-quality wheat cultivars (Wang et al., 2022). Association analysis is based on linkage disequilibrium (LD) and enables the detection and mapping of candidate genes controlling target traits within a population. Compared with linkage analysis, GWAS provides higher mapping resolution, reduces time requirements by exploiting natural populations, and captures high levels of polymorphism, allowing multi-trait analysis and the dissection of complex traits at the species level (Remington et al., 2001; Breseghello and Sorrells, 2006; Rasheed et al., 2014; Zanke et al., 2015; Bilgrami et al., 2020; Kassem, 2025).

Although numerous GWAS studies have been conducted on wheat yield-related traits, most have focused on a limited set of traits or specific growth stages, leaving a comprehensive understanding of the genetic architecture across both seedling and adult stages largely unexplored. Furthermore, previous studies have often employed relatively low-density marker platforms or narrow genetic backgrounds, resulting in limited power to detect stable, environmentally robust loci. The hexaploid wheat genome is exceptionally large (~16 Gb) with high sequence similarity among homologous subgenomes and over 85% repetitive DNA (Martín et al., 2018), which has historically constrained GWAS applications. Only after the release of the Chinese Spring reference genome in 2018 did the genomic foundation for wheat GWAS become widely accessible. However, systematic analyses integrating multiple yield-related traits across different growth stages within a single diverse population remain scarce.

The novelty of this study lies in three aspects: (1) A large natural population comprising 768 wheat accessions was used to systematically investigate 14 yield-related traits covering both seedling and adult stages, which enables a more integrated dissection of the genetic basis underlying wheat yield formation. (2) The high-density 55K SNP array was applied to improve the resolution and reliability of genome-wide association mapping, facilitating the detection of stable and robust genetic loci. (3) Multiple pleiotropic loci simultaneously associated with different traits were identified, which helps reveal the genetic correlation among various yield-related traits and provides valuable genetic resources and molecular markers for wheat molecular breeding.

In this study, we evaluated a natural wheat population of 768 varieties and lines. Hydroponic cultivation was used to assess seedling traits, including root length, seedling height, and seedling weight. At the adult stage, plant height, spike-related traits, and other yield-related traits were measured. The population was genotyped using a wheat 55K SNP array, followed by GWAS of 14 yield-related traits. The objectives were to identify SNP markers and candidate genes significantly associated with major traits across growth stages, to uncover relevant alleles and loci, and to facilitate the exploration and utilization of valuable genetic resources in common wheat. A key strength of this study is the simultaneous analysis of 14 traits within a single diverse population using a high-density SNP array, enabling the detection of pleiotropic loci. However, several limitations should be acknowledged: the 55K SNP array may not capture all structural variants or rare alleles; the natural population structure, while reflecting real-world diversity, may introduce spurious associations requiring careful statistical correction; and candidate gene identification from GWAS signals is predictive and requires subsequent functional validation. Despite these limitations, the robust associations identified across two years and the detection of multi-trait loci provide a solid foundation for downstream gene cloning and marker-assisted selection.

## 2 Materials and methods

### 2.1 Headings

The plant materials comprised a diverse panel of 768 wheat (*Triticum aestivum* L.) accessions collected from 28 countries and regions (**Supplementary Table 1**). The panel included landraces and introduced materials (38%), with the remaining 62% comprising advanced breeding lines and released cultivars. Together, these materials provided broad genetic representation and substantial diversity for association mapping. Field experiments were performed at the Shandong Agricultural University Agronomy Experimental Station in Tai’an from 2023 to 2025. The field experiment followed a randomized complete block design with two replications. Each genotype was sown in five-row plots, with 40 seeds per row. Rows were 2 m long with 25 cm spacing. Eleven major agronomic traits, excluding seedling-stage traits, were evaluated under field conditions. These included heading stage (HS), flowering stage (FS), maturity period (MP), plant height (PH), spike length (SL), tiller number (TN), grain number per spike (GNS), total spikelet number per spike (TSS), kernel number per spikelet (KNS), sterile spikelet number per spike (SSS), and grain yield (GY).

### 2.2 Hydroponic seedling culture

For hydroponic culture, ten intact and uniform seeds per accession were surface-sterilized with 10% (v/v) H_2_O_2_ for 5 min and thoroughly rinsed with distilled water. Seeds were placed into 1.5 cm diameter holes of hydroponic boards lined with gauze at the base, with each board containing 120 holes. The boards were set in plastic boxes measuring 1.2 m × 0.6 m, each filled with 8 L of Hoagland nutrient solution (**Table 1**). Oxygen was continuously supplied using an ACO-007 electromagnetic air pump (power: 120 W, air pressure: 0.04 MPa, exhaust volume: 90 L/min). After germination, seedlings were thinned, and five plants per line with uniform growth and vigor were retained for root analysis. The growth conditions were maintained at a 16 h light/8 h dark photoperiod, temperatures of 18–20 °C, and relative humidity above 60%. After 20 days of hydroponic cultivation, root systems were collected simultaneously, fixed in Formalin-Aceto-Alcohol (FAA), and used for seedling root phenotypic evaluation.

**Table 1:** Nutrient solution ingredients for wheat seedling growth in control treatment.

Macronutrients	Concentration (mmol/L)	Trace elements	Concentration (μmol/L)
KH_2_PO_4_	0.2	H_3_BO_3_	1
MgSO_4_·7H_2_O	0.5	(NH_4_)_6_Mo_7_O_24_·4H_2_O	0.1
KCl	1.8	CuSO_4_·5H_2_O	0.5
CaCl_2_	1.5	ZnSO_4_·7H_2_O	1
(NH_4_)_2_SO_4_·H_2_O	1	MnSO_4_·H_2_O	1
Ca(NO_3_)_2_·H_2_O	1	Fe·EDTA	100

### 2.3 Seedling-stage trait investigation

Root Length (RL): Root length was defined as the distance from the base of the root to the root tip. Extracted wheat root systems were rinsed with tap water and subsequently washed twice with deionized water. The maximum root length per plant was measured using a ruler, with three biological replicates per accession.

Seedling Height (SH): Seedling height was defined as the distance from the base of the root to the leaf tip. Height per plant was measured using a ruler, with three biological replicates per accession.

Seedling weight (SW): Seedling weight was defined as the fresh weight of all leaves. Fresh weight was determined using a balance, with three biological replicates per accession.

### 2.4 Investigation methods for 11 phenotypic traits

HS, FS, MP were recorded in the field during the corresponding growth stages. For PH, SL, TN, ten plants were randomly selected from each plot and measured in the field. At harvest, ten main stems were randomly collected from each plot and brought to the laboratory to measure GNS, TSS, KNS, SSS. GY was determined by harvesting the whole plot, followed by threshing and weighing. The measurement methods for these traits are detailed in **Table 2**.

**Table 2:** Summary of investigated traits and their measurement methods in wheat.

Abbreviations	Traits	Units	Methods of measurement
HS	Heading stage	d	More than 1/2 of spikes emerged indicates entry into the heading stage
FS	Flowering stage	d	More than 1/2 of spikes flowered indicates entering the flowering stage
MP	Mature period	d	Most grains become hard, size and color close to normal, not easily scratched by fingertip
PH	Plant height	cm	Plant height of the main stem per wheat plant
SL	Spike length	cm	Length of the main spike per wheat plant
TN	Tiller number	pcs	Number of tillers per wheat plant
GNS	Grain number per spike	kernel	Number of grains in the main spike per wheat plant
TSS	Total spikelet number per spike	pcs	Total number of spikelets in the main spike per wheat plant
KNS	Kernel number per spikelet	kernel	Average number of grains per spikelet in the main spike
SSS	Sterile spikelet number per spike	pcs	Number of sterile spikelets in the main spike (BSSS + TSSS)
GY	Grain yield	g	Plot yield measured after harvest

### 2.5 Genomic DNA extraction and SNP genotyping

Genomic DNA was extracted from seedling leaves of each accession using the cetyl trimethyl ammonium bromide method (Stewart and Via, 1993). Genome-wide genotyping was conducted using the Wheat 55K SNP array (CapitalBio Technology, Beijing, China), which assays 53,063 putative SNP loci.

### 2.6 Population genetic structure analysis

First, 45,298 SNPs with a missing rate ≤ 0.5 and a minor allele frequency (MAF) ≥ 0.05 were retained from an initial set of 53,063 SNPs using an in-house Python script. Subsequently, linkage disequilibrium (LD) pruning was performed using PLINK (Purcell et al., 2007), with a window size of 50, step size of 50, and an r² threshold of 0.3, resulting in 4,360 independent SNPs. Population structure was inferred using STRUCTURE v2.3 across K values from 1 to 20 (Pritchard et al., 2000; Evanno et al., 2005). Concurrently, principal component analysis (PCA) and kinship estimation were conducted using the R package GAPIT. The population structure and final number of subpopulations were determined by integrating the results from these three analyses (Lipka et al., 2012; Tang et al., 2016).

### 2.7 Phenotypic data processing

Phenotypic data from 768 wheat accessions were statistically analyzed using Microsoft Excel 2019. In this study, best linear unbiased prediction (BLUP) values for each trait were calculated using the lmer function from the lme4 package (version 1.1-35) in R. All effects except the intercept were treated as random effects. The model structure was specified as follows:


$$Y_{ijk}=\;\mu +\;G_{i}+\;E_{j}+\;{\left(G\;\times E\right)}_{ij}+\;R{\left(E\right)}_{jk}+\;\epsilon_{ijk}$$

where: *Y_ijk_* represents the phenotypic value of the *i*-th genotype in the *j*-th environment for the *k*-th replicate; *μ* is the overall mean (fixed effect); *G_i_* is the effect of the *i*-th genotype (random); *E_j_* is the effect of the *j*-th environment (random); (*G*×*E*)*_ij_* is the genotype-by-environment interaction effect (random); *R*(*E*)*_jk_* is the replicate effect nested within environment (random); *ϵ_ijk_* is the residual error (random). BLUP values for each genotype were extracted using the coef(fm)$Code function and subsequently used for GWAS.

### 2.8 Genome-wide association study

Association analyses for 14 traits were performed separately for each year using GAPIT. A linear mixed model (LMM) incorporating principal component (PC) values was applied. The significance threshold for genome-wide association was determined through conditional permutation tests with 1000 replications. The conditional permutation test was executed using LMM with the same parameters and PC matrix. SNPs with -log(*P*) > 2 in the GWAS using original phenotypes were extracted to improve the computational efficiency. Finally a threshold of -log(*P*) = 3.64 was determined, which was higher than the 95th percentile of 1,000 conditional permutation tests (Zhao et al., 2018).

## 3 Results

### 3.1 Screening of superior germplasm

A total of 12 wheat germplasm materials with superior yield and quality were identified from 768 accessions, with Jimai 22 used as the control cultivar (**Table 3**). These elite lines exhibited a GY advantage of ≥ 3.91%, a thousand-grain weight above 44 g. Their targeted utilization provides a strong foundation for breeding new wheat varieties that integrate high yield, high quality, and early maturity.

**Table 3:** Selection of excellent wheat germplasm materials.

Material	Yield per Mu (kg)	Increase rate (%)	Thousand-grain weight (g)
SN B35-1	578.7	3.95	52.03**
BC15PTJ115	586.7	5.15	47.15**
SN B25	594.7	6.78	51.72**
SN 15	592.0	6.29	53.32**
15-5340	589.4	6.00	51.63**
SN 542921	602.7	8.43	51.33**
SN 2149	586.7	5.31	46.40**
JM 41753	626.7*	12.55	53.87**
TM 33	578.7	3.91	47.02**
15-6327	613.4*	10.11	49.53**
15-5437	610.7*	9.48	51.82**
Ta14-7022	608.0*	9.08	51.12**
JM 22	557.4	–	44.11

Yield per Mu and Thousand-grain weight are BLUP values. T-tests were performed using field-measured data, with each accession compared to the control (Jimai 22); 0.001 ≤**p < 0.01.

### 3.2 Descriptive statistics of wheat traits

Descriptive statistical analyses were conducted on data from 768 wheat accessions, covering three seedling-stage traits and 11 phenotypic traits evaluated over two consecutive years. Significant variation was detected in all seedling-stage traits, including seedling weight (SW), root length (RL), and seedling height (SH), with coefficients of variation (CVs) exceeding 20%. Among them, SW showed the greatest variability (CV = 24.9%), followed by RL (21.9%) and SH (21.4%). For the eight major agronomic traits, first-year CVs ranged from 1.4% to 75.9%. SSS displayed the highest variability, whereas MP showed the lowest. A comparable trend was observed in the second year, with CVs ranging from 1.5% to 61.8%, again with SSS highest and MP lowest. Across both years, mean CVs spanned from 1.4% for MP to 57.4% for SSS, indicating rich phenotypic diversity within the population. By contrast, the growth-stage traits (HS, FS, and MP) exhibited limited variation, with two-year mean CVs of 1.9%, 1.8%, and 1.4%, respectively. This narrow variability indicates strong local adaptation in modern wheat cultivars, making it difficult for breeders to develop substantially earlier-maturing varieties (**Table 4**).

**Table 4:** Descriptive statistics of 11 phenotypic traits.

Trait	2023–2024				2024–2025				AV				*p*-value
	Mean	Min	Max	CV (%)	Mean	Min	Max	CV (%)	Mean	Min	Max	CV (%)	
HS	188.2	182	209	1.8	197.9	189	214	1.9	192.7	185.5	211.5	1.9	< 0.001***
FS	192.7	186	213	1.8	203.0	194	217	1.6	197.5	190.0	215.0	1.8	< 0.001***
MP	229.9	223	244	1.4	233.6	225	261	1.5	231.6	225.5	245.0	1.4	< 0.001***
PH	75.2	37.8	130	21.8	85.2	38	150	22.5	79.8	38.9	138.5	21.4	< 0.001***
SL	8.7	5.2	14.8	18.3	9.8	5.87	19.65	18.1	9.2	6.2	15.8	16.7	< 0.001***
TN	14.9	6.5	38.5	34.1	16.7	3.7	43	30.1	15.8	7.6	32.5	24.0	< 0.001***
TSS	18.2	11.7	31.2	13.7	20.7	11.1	32.3	10.4	19.3	13.6	31.8	10.4	< 0.001***
SSS	0.8	0.1	3.2	75.9	1.1	0.1	4.4	61.8	0.9	0.1	4.4	57.4	0.946
KNS	4.0	2.6	6.4	14.4	3.9	2.3	5.4	12.8	3.9	2.6	5.7	11.8	< 0.001***
GNS	51.3	28	107	21.1	57.8	31.3	93.8	15.5	54.3	32.0	93.6	15.6	< 0.001***
GY	411.8	21.33	709.37	29.8	429.8	22.22	1057.83	33.8	420.0	32.0	742.3	27.5	< 0.001***

AV, Average; HS, Heading stage, FS, Flowering stage, MP, Mature period, PH, Plant height, SL, Spike length, TN, Tiller number, GNS, Grain number per spike, TSS, Total spikelet number per spike, KNS, Kernel number per spikelet, SSS, Sterile spikelet number per spike, GY, Grain yield; ***p < 0.001, no asterisks = non-significant.

### 3.3 Statistical analysis of wheat traits

**Figure 1:**
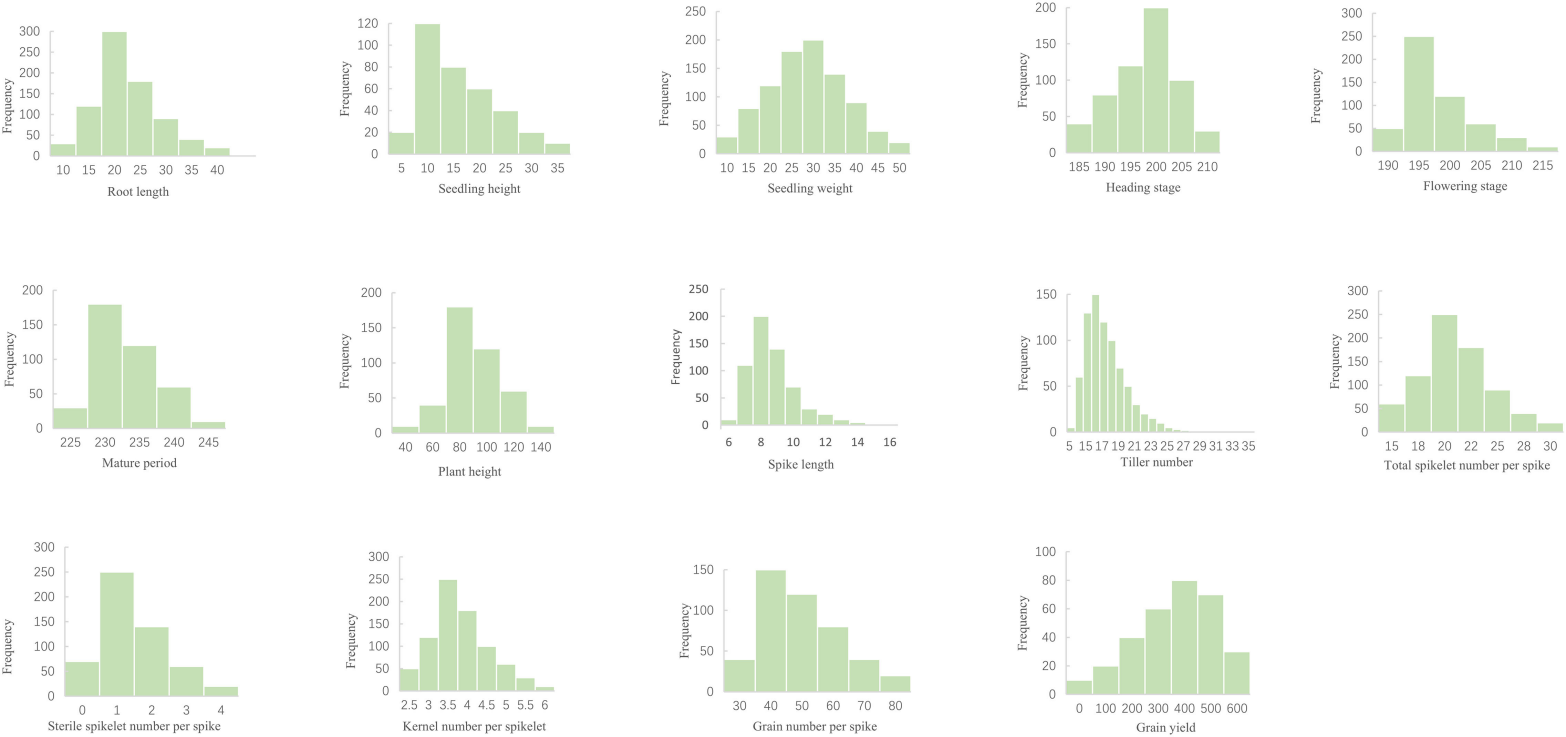
Frequency distributions of 14 yield-related traits. Histograms showing the phenotypic distribution of 14 yield-related traits measured across 768 wheat accessions. The x-axis represents trait values, and the y-axis indicates the number of accessions.

Phenotypic variation was further analyzed for 14 yield-related traits in the same natural population (**Figure 1**). RL, SW, KNS, and TSS followed normal distributions, consistent with their polygenic quantitative inheritance. In contrast, GY, SL, TN, HS, FS, SSS, MP, and PH exhibited varying degrees of skewness. Although these traits remain quantitative, the observed distribution patterns suggest the involvement of genes with major effects. Notably, SSS, MP, and PH showed the strongest skewness, supporting the presence of prominent genetic factors controlling these traits.

### 3.4 Correlation analysis of wheat traits

Pearson correlation analysis was conducted for 14 yield-related traits, including three seedling-stage traits, across 768 wheat accessions. Correlation strength was classified according to the absolute value of Pearson’s r as follows: 0.8–1.0 (very strong), 0.6–0.8 (strong), 0.4–0.6 (moderate), 0.2–0.4 (weak), and 0.0–0.2 (very weak or none). Statistical significance was evaluated for all trait correlations.

Among the 105 trait pairs examined, 50 (47.6%) showed significant correlations, including 44 correlations significant at P < 0.01 and 6 correlations significant at P < 0.05 (**Supplementary Table 2**). These findings revealed a complex network of both positive and negative trait associations, highlighting yield as an integrative trait shaped by multiple interacting and competing components. Therefore, effective yield improvement requires the simultaneous consideration of multiple traits and their interrelationships to achieve an optimal balance.

### 3.5 SNP polymorphism analysis and population structure

**Figure 2:**
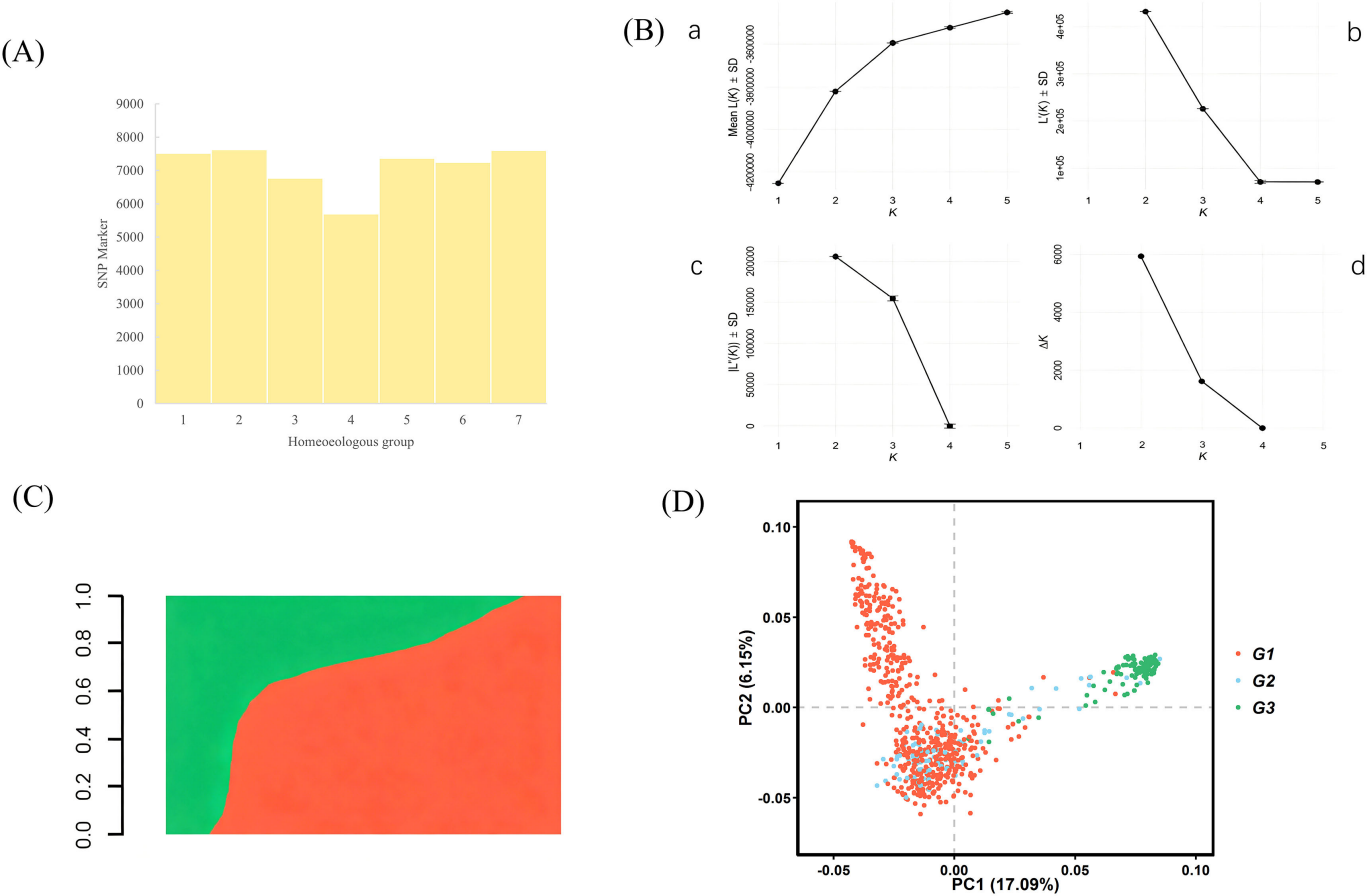
SNP polymorphism analysis and population structure of the 768 wheat accessions. **(A)** distributions of polymorphic SNPs on individual chromosome and each homoeologous group. **(B)** determination of the optimal number of subpopulations (K) using STRUCTURE. (a, mean L(K) ± SD of STRUCTURE runs for K values from 1 to 5; b, The first-order rate of change of the likelihood function, L’(K); c, the absolute value of the second-order rate of change of the likelihood function, |L’’(K)|; d, The ΔK values for each (K) The peak at K = 2 indicates the optimal number of subpopulations). **(C)** STRUCTURE analysis of the wheat panel at K = 2. Green: local wheat varieties; red: modern improved varieties. **(D)** principal component analysis (PCA) of the 768 wheat accessions based on genome-wide SNPs. The first two principal components, PC1 and PC2 are plotted, with the percentage of variance explained shown in parentheses.

The association panel was genotyped using the Wheat 55K SNP array, which comprises 53,063 high-quality SNP markers. After filtering for MAF ≥ 5% and a missing rate ≤ 50%, 49,768 high-quality SNP markers were retained for subsequent analyses. These markers provided an average genome-wide coverage of 82.73%, ranging from 66.41% to 90.64%. Marker distribution was uneven across the A, B, and D subgenomes, which contained 17,293, 18,313, and 14,162 SNPs at mean physical intervals of 0.29 Mb, 0.28 Mb, and 0.31 Mb, respectively (**Figure 2A**). Similarly, SNP density across the seven homoeologous groups was uneven, with the order ranked as: 2 > 7 > 1 > 5 > 6 > 3 > 4 (**Supplementary Table 3**). LD decay analysis was performed using Plink with a window size of 50, step size of 50, and r^2^ ≥ 0.3, resulting in the identification of 4,360 independent SNP loci. Population structure was inferred using STRUCTURE, where the true K value was determined based on the modal value of LnP(D). A pronounced ΔK peak at K = 2 indicated that the 768 accessions were clearly divided into two groups (**Figure 2B**). Consistent with this result, kinship analysis also resolved two distinct subgroups corresponding to original local wheat varieties and modern improved varieties (**Figure 2C**). Principal component analysis showed that the first principal component explained 17.09% of the total genetic variation, while the first three principal components collectively accounted for 26.62% (**Figure 2D**). Together, these results demonstrate clear genetic differentiation between endemic bread wheat landraces and improved cultivars.

### 3.6 Genome-wide association analysis of 3 seedling-stage traits in wheat

A GWAS was conducted for three seedling-stage traits, SH, RL, and SW, using 49,768 high-quality SNP markers with a missing rate ≤ 50% and MAF ≥ 0.05. To control false positives caused by population structure, a linear mixed model (LMM) was applied. This analysis identified 74 significant SNP–trait associations, mainly distributed on chromosomes 1A, 3A, 3D, and 6D (**Supplementary Table 4**).

For RL, 41 significant SNPs were identified, with 32 SNPs clustered on chromosome 6D. This interval contained 33 high-confidence genes based on the Chinese Spring reference genome (CS RefSeq v1.1). Among them, several genes were functionally related to root development, signal transduction, and stress responses, including a LOB domain protein-like gene (*TraesCS6D02G382600*), an ethylene receptor gene (*TraesCS6D02G383600*), a receptor-like kinase gene (*TraesCS6D02G383700*), and several leucine-rich repeat receptor-like protein kinase and NBS-LRR disease-resistance genes.

For SH, 21 significant SNPs were detected, including 12 markers located on chromosome 3A. This region contained six high-confidence genes, among which three encoded cytochrome P450-related proteins (*TraesCS3A02G152500*, *TraesCS3A02G152700*, and *TraesCS3A02G153000*), suggesting potential roles in hormone metabolism, secondary metabolism, or oxidative stress responses.

For SW, 12 significant SNPs were identified, including seven clustered on chromosome 1A. This interval harbored 69 high-confidence genes, including genes encoding transcription factors, kinases, protein phosphatase 2C, transporters, detoxification-related proteins, chitinases, glutathione S-transferase, metallothionein, and ubiquitin-related proteins. These genes may participate in seedling biomass accumulation through regulation of stress response, transport, protein modification, and metabolic processes (**Supplementary Table 5**).

**Figure 3:**
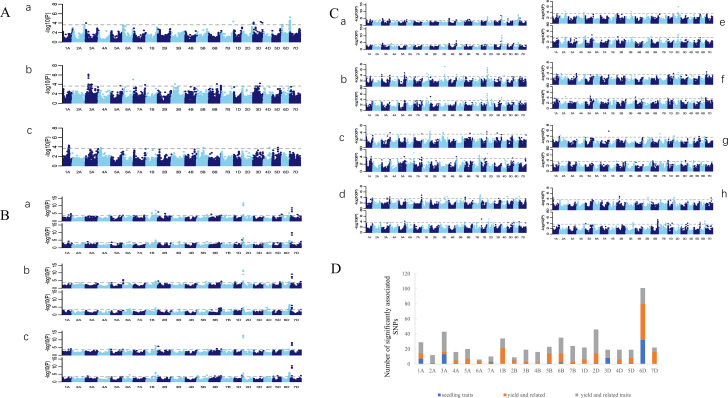
Genome-wide association study (GWAS) results for 14 yield-related traits. **(A)** Manhattan plots for three seedling-stage traits from GWAS (a: root length; b: seedling height; c: seedling weight). **(B)** Manhattan plots for three growth-stage traits from GWAS (a, heading stage; b, flowering stage; c, mature period). **(C)** Manhattan plots for eight major agronomic traits from GWAS (a, plant height; b, spike length; c, tiller number; d, grain number per spike; e, total spikelet number per spike; f, kernel number per spikelet; g, sterile spikelet number per spike; h, grain yield). In all Manhattan plots, the x-axis shows the physical position on each chromosome, and the y-axis represents –log(*P*) values from the linear mixed model. The horizontal dashed line indicates the genome-wide significance threshold. **(D)** Chromosomal distribution of SNPs significantly associated with the 14 traits. Different colors represent different trait categories.

No SNP markers were commonly associated with all three traits, suggesting that SH, RL, and SW may be largely controlled by distinct loci at the seedling stage. Protein sequence alignment with rice provided additional support for candidate gene prioritization. Notably, the rice homolog *Os02g0820500*, corresponding to the RL-related gene *TraesCS6D02G382600*, has been reported to regulate crown root development, suggesting that *TraesCS6D02G382600* is a promising candidate gene for root length variation (**Supplementary Table 6**; **Figure 3A**).

### 3.7 Genome-wide association analysis of 11 phenotypic traits in wheat

Association analysis of three growth-stage traits, FS, HS, and MP, across two years identified 176 significant SNP markers, of which 27 were consistently detected in both years. These markers were mainly distributed on chromosomes 1B, 5B, 6B, 2D, 6D, and 7D, with no associations detected on chromosomes 2A or 3D (**Supplementary Table 7**; **Figure 3B**). The smallest associated genomic interval spanned 0.175 Mb and contained only three high-confidence genes. To further identify candidate genes, high-confidence genes within the significant SNP-associated intervals were annotated using the Chinese Spring reference genome (CS RefSeq v1.1). For FS, HS, and MP, several genes were predicted to be involved in flowering regulation, developmental transition, signal transduction, and stress response, including genes encoding nuclear transcription factor Y protein, MADS-box transcription factor, pseudo-response regulator, AP2-like ethylene-responsive transcription factor, TCP transcription factor, NAC domain-containing protein, cytochrome P450, ascorbate peroxidase, F-box protein, RING/U-box protein, and GDSL esterase/lipase. Protein sequence alignment with rice provided additional support for candidate gene prioritization (**Supplementary Table 8**). The rice homologs *Os07g0695100* and *Os07g0694700*, corresponding to *TraesCS2D02G079600* and *TraesCS2D02G080000*, were associated with HS. The rice homolog *Os05g0494100* of *TraesCS1B02G322000* was related to FS. For MP, *Os02g0747900*, *Os02g0746000*, and *Os06g0156600*, corresponding to *TraesCS6D02G285300*, *TraesCS6D02G285800*, and *TraesCS7D02G108900*, respectively, were associated with maturity stage (**Supplementary Table 9**).

For the eight major agronomic traits, significant SNPs were detected on all chromosomes, with pronounced enrichment on chromosomes 3A, 6B, 7B, 2D, and 6D. Among these, 49 loci were consistently significant across both years, providing promising targets for further breeding application and functional characterization (**Supplementary Table 10**; **Figure 3C**). For PH, SL, and TSS, high-confidence genes within significant SNP-associated intervals were identified and functionally annotated. Candidate genes for PH were mainly related to hormone signaling, transcriptional regulation, and kinase signaling, including genes encoding auxin-responsive protein, MYB-related transcription factor, nuclear transcription factor Y subunit, ethylene receptor, ethylene-insensitive protein 2, MAP kinase, TEOSINTE BRANCHED 1, and GAI-like protein. For SL and TSS, candidate genes were mainly involved in spike development, signal transduction, protein modification, and metabolism, including receptor kinase, NAC domain protein, response regulator, F-box protein, ubiquitin-related protein, cytochrome P450, 2-oxoglutarate/Fe(II)-dependent oxygenase, and RING domain ligase (**Supplementary Table 3**; **Figure 3D**). Four SNPs on chromosome 2D, namely Chr2D_23102495, Chr2D_23304055, Chr2D_23416184, and Chr2D_37041200, were concurrently associated with PH and SL. Additionally, 20 SNPs showed significant associations with all three growth-stage traits HS, FS, and MP. Of these, seven were located on chromosome 6D, four on chromosome 7D, three on chromosome 2D, two on chromosome 1B, one on chromosome 1D, one on chromosome 2B, one on chromosome 6A and one on chromosome 6B. Six loci were consistently associated with all three traits in both years, including Chr1B_545202367, Chr2D_32795283, Chr2D_32970798, Chr7D_65503524, Chr7D_66537887, and Chr7D_67448018. The identification of pleiotropic SNP loci is highly valuable for genetic dissection and breeding applications. These markers enable the simultaneous improvement of multiple traits, thereby enhancing breeding efficiency. The stable multi-trait-associated SNPs identified in this study represent promising candidates for conversion into molecular markers for marker-assisted selection, facilitating the concurrent screening of multiple desirable traits in breeding programs.

## 4 Discussion

### 4.1 Application value of elite parents

Germplasm resources serve as the foundation for wheat breeding. In conventional breeding programs, breeders have long focused on the selection and development of elite parents, which serve as the basis for new variety development (Krishna et al., 2023). These elite parents are typically characterized by superior agronomic traits, strong disease resistance, abundant favorable alleles, high combining ability, and high heritability, and have been widely used in the development of new wheat varieties. However, the prolonged and intensive use of a limited set of elite parents has gradually narrowed the genetic basis of modern wheat cultivars, thereby limiting further breakthroughs in breeding potential and increasing the vulnerability of varieties to emerging pathogen races and environmental stresses (Lehnert et al., 2022; Singh et al., 2021). Therefore, the systematic exploration and utilization of diverse germplasm resources to broaden the genetic foundation of elite parents has become an urgent challenge in wheat breeding. In this study, 768 wheat accessions originating from different wheat-growing regions and representing various types were selected for systematic investigation (**Supplementary Table 1**). Agronomic traits were evaluated and subjected to comparative analysis, association mapping, and targeted screening. By analyzing the accessions based on population structure and geographic origin, this study aims to identify valuable germplasm resources—including potential elite parents—for different ecological regions. The findings are expected to provide a scientific basis for trait improvement and breeding practices tailored to specific ecological zones.

### 4.2 Formation of the population structure of common wheat

Landraces of common wheat generally harbor higher genetic diversity than modern improved cultivars, largely due to the intensive selection and widespread use of a limited set of elite germplasm, such as the semi-dwarf, photoperiod-insensitive lines underlying the “Green Revolution” (Tang et al., 2023; Sansaloni et al., 2020; Hedden, 2003; Ma et al., 2019b). Although introgression from wild relatives is known to contribute valuable genetic variation, systematic understanding of how these introgressions shape subpopulation differentiation and the genetic architecture of modern varieties remains limited. In this study, 768 wheat accessions were genotyped using a 55K SNP array. Population structure and kinship analyses clearly distinguished two major groups: primitive landraces and modern improved cultivars, confirming significant genetic differentiation between these germplasm types (**Figures 2B–D**). However, this study primarily describes basic population structure without dissecting the fine-scale subpopulation architecture, the specific genomic regions driving differentiation, or their links to agronomic traits. Further investigation into the detailed population structure of different germplasm types is needed to guide parental selection and accelerate variety development.

### 4.3 Mining of wheat important trait-related QTL genes

QTL mapping remains a primary approach for identifying genetic loci controlling target traits in wheat populations. Yield-related QTLs are unevenly distributed across chromosomes, with higher densities on chromosomes 4B, 5A, and 7A, and fewer loci on 1D, 6B, and 6D. Most reported QTLs are associated with thousand-grain weight (Kiseleva et al., 2023; Cao et al., 2020). With the widespread use of SNP chips and sequencing-based genotyping platforms, association mapping has become an important strategy for QTL discovery in wheat (Tong et al., 2024). Although several loci now serve as useful references in breeding, the identification and functional characterization of their candidate genes require further strengthening. Previous studies have identified numerous regulatory genes underlying key agronomic traits, providing valuable genetic targets for wheat improvement, and the present findings are consistent with this progress. Continued mining and validation of candidate genes within these QTL regions are therefore expected to be a major focus of future research, supporting functional marker development, marker-assisted selection, and gene-editing-based functional validation.

In this study, we compared the 544 significantly associated SNPs with previously reported QTLs and functional genes. On chromosome 2D (23.1–37.0 Mb), SNPs significantly associated with spike length, spikelet number, and plant height colocalized with the pleiotropic locus *QPht/Sl/Sn.cau-2D.1* and the cloned gene *TaRGN-D1* (Chai et al., 2018; Jiang et al., 2025). The major QTL *QSns.sau-2D* was also confirmed (Ma et al., 2019a). On chromosome 6D, the root length QTL *qRS-6D* was independently validated (Cheng et al., 2024). We further identified *TraesCS6D02G382600* as a novel candidate gene for this locus, which has not been previously reported to be associated with root development in wheat. On chromosome 7DS, SNPs associated with heading stage colocalized with two functionally validated flowering-time genes, *TaFT-D1* and *TaSEP3-D1*, as well as the classical earliness per se QTL *Eps-7D* (Chen et al., 2022; Li et al., 2026; Basavaraddi et al., 2021). On chromosome 4D, the classical dwarfing gene *Rht-D1b* was further validated by plant-height-associated SNPs (Peng et al., 1999). Notably, more than 80% of the significant SNPs identified in this study were not found to overlap with previously reported wheat GWAS or QTL mapping results, and these novel loci and their associated alleles represent valuable genetic resources for wheat genetic improvement. The rice orthologs of fifteen candidate genes within our SNP-enriched intervals have been cloned and functionally characterized in rice (**Supplementary Tables 6, 9, 12**). For example, the rice ortholog of *TraesCS6D02G382600*, *Os02g0820500*, regulates crown root development, providing a high-priority candidate for subsequent functional validation and marker development in wheat.

### 4.4 Pleiotropy

Genetic pleiotropy refers to the ability of a single gene or genetic variant to influence two ormore phenotypic traits simultaneously (Mehra and Hintze, 2024). Because many biochemical pathways are interconnected during organismal development, a gene regulating one trait may also affect multiple downstream processes. Consequently, in molecular breeding, genetic analyses that focus on a single sub-trait are often insufficient for improving complex traits. For example, wheat yield per mu is jointly determined by the number of spikes per mu, kernel number per spike, and thousand-grain weight, which are typically interrelated and exhibit genetic co-variation. In wheat production, varieties with excessively high thousand-grain weight frequently show reduced kernel number per spike and fewer spikes per mu (Klingenberg, 2008; Fang et al., 2017). Such trait correlations may arise from pleiotropy, linkage disequilibrium among causal loci, or developmental and physiological interdependence, although their underlying genetic basis remains debated, although the underlying genetic basis remains debated (Chen and Lübbenstedt, 2010). Previous studies have distinguished between tightly linked loci and pleiotropic loci. Although a gene is considered the smallest genetic unit, pleiotropic effects may arise from linkage among multiple quantitative trait nucleotides (QTNs) within a single gene, with each QTN influencing a different trait. In contrast, true pleiotropy occurs when a single QTN affects two or more traits (Zhang et al., 2018). Accordingly, pleiotropic genes can be classified into two types. The first type comprises genes with a single function that participate in multiple biological processes. For instance, the wheat flowering regulator *TaFT-D1* also affects grain weight by interacting with the positive grain weight regulator *TaFDL2*, thereby co-activating downstream genes involved in cell cycle and starch biosynthesis (Zhang et al., 2025). The second type consists of genes with multiple functions that contribute to different traits. An example is the Green Revolution gene *Rht-D1b*, which, in addition to reducing plant height, also regulates tiller angle and tiller number (Zhang et al., 2026b). The effective use of pleiotropic genes in breeding remains challenging because favorable and unfavorable traits are often genetically coupled.

In the present study, a total of 60 SNP markers were significantly associated with two or more agronomic traits. Notably, these 60 multi-trait-associated loci were defined at the marker level as identical SNPs significantly associated with multiple traits, thereby excluding cases in which different SNPs within the same LD block were separately associated with distinct traits (**Supplementary Table 13**). However, it is important to note that several agronomic traits in wheat, such as plant height and spike length, as well as multiple growth-stage-related traits, display strong phenotypic and statistical correlations. Consequently, for such highly correlated traits, the shared SNP associations may merely reflect statistical correlations among the traits rather than genuine biological pleiotropy. Therefore, while these 60 SNPs indicate potential pleiotropic effects, caution is needed in their interpretation. Additional analyses, such as conditional GWAS, fine mapping, or functional validation, are required to distinguish true pleiotropy from linkage disequilibrium and trait correlation. Even so, the repeated detection of identical SNP markers across multiple traits provides evidence for widespread multi-trait genetic associations during the wheat growth cycle. Collectively, these findings suggest that these loci may harbor functional variants with broad regulatory effects on wheat agronomic performance, providing a foundation for future mechanistic studies and breeding applications.

## 5 Conclusion

This study elucidates the genetic architecture of yield-related traits in a diverse panel of 768 common wheat cultivars. By integrating high-density genotyping with multi-environment phenotyping, we identified 544 significant marker–trait associations, including 49 environmentally stable loci and 60 with putative pleiotropic effects. Clear genetic differentiation was observed between modern cultivars and landraces, reflecting their divergent evolutionary paths and offering novel germplasm and allelic diversity for future breeding. Future efforts should focus on fine mapping and functional validation of stable loci, mechanistic dissection of pleiotropic effects, exploration of untapped diversity in landraces, and integration with pan-genomic and machine learning approaches to accelerate breeding.

## Data Availability

All relevant data is contained within the article: The original contributions presented in the study are included in the article/**Supplementary Material**, further inquiries can be directed to the corresponding author/s.
